# Draft Genome Sequence of *Sphingobium quisquiliarum* Strain P25^T^, a Novel Hexachlorocyclohexane (HCH)-Degrading Bacterium Isolated from an HCH Dumpsite

**DOI:** 10.1128/genomeA.00717-13

**Published:** 2013-09-12

**Authors:** Amit Kumar Singh, Naseer Sangwan, Anukriti Sharma, Vipin Gupta, J. P. Khurana, Rup Lal

**Affiliations:** Department of Zoology, University of Delhi, Delhi, Indiaa; Interdisciplinary Centre for Plant Genomics & Department of Plant Molecular Biology, University of Delhi South Campus, New Delhi, Indiab

## Abstract

Here, we report the draft genome sequence (4.2 Mb) of *Sphingobium quisquiliarum* strain P25^T^, a natural *lin* (genes involved in degradation of hexachlorocyclohexane [HCH] isomers) variant genotype, isolated from a heavily contaminated (450 mg HCH/g of soil) HCH dumpsite.

## GENOME ANNOUNCEMENT

The disposal of hexachlorocylohexane (HCH) waste in the past has resulted in the pangenomic enrichment of various sphingomonad genotypes at HCH dumpsites (1, 2). In order to continue our efforts to sequence genomes of sphingomonads from the HCH dumpsite located near Lucknow, India (27°00′N and 81°09′E) (3, 4), we sequenced the genome of another sphingomonad strain, P25^T^ (4.2 Mb).

The draft genome sequence of strain P25^T^ was obtained by use of an Illumina Genome Analyzer II platform. The sequencing data (*n* = 3,882,670; 90 bp/read) were assembled into contigs (*n* = 181, >500 bp) using ABySS 1.3.3 (5) set at a k-mer size of 47. Contigs (*N*_50_, 45 kb) were further validated (paired-end criterion) using bwa-0.5.9 (6). Glimmer-3.02 (7) was used to predict the protein-encoding genes, whereas tRNA and rRNA genes were identified using ARAGORN (8) and RNAmmer (9), respectively. A total of 4,033 coding sequences (CDS), 70 pseudogenes, 54 tRNA genes, and 1 rRNA operon were observed, with an average G+C content of 64%. Validated (paired-end criterion) genome assembly was annotated using RAST version 4.0 (10) and the NCBI Prokaryotic Genomes Automatic Annotation pipeline (PGAAP) (http://www.ncbi.nlm.nih.gov/genomes/static/Pipeline.html). Average nucleotide identity (ANI) (11) analysis revealed that *Sphingobium japonicum* UT26S (83.3%) (12), *Sphingobium indicum* B90A (83.0%) (4), and *Sphingomonas* sp. SKA58 (80.8%) are the closest phylogenetic neighbors of *S. quisquiliarum* P25^T^.

The mechanisms of acquisition of *lin* genes in sphingomonads under HCH stress at these dumpsites are still not clearly understood (2). The *lin* genes were first reported in *S. japonicum* UT26 (12) and subsequently from *S. indicum* B90A (4). Many more sphingomonads have been isolated recently from the HCH dumpsite (2, 14). All of these strains by and large share the same pathway for the degradation of HCH isomers that requires the *linA* through *linF* genes (2). Interestingly, the analysis of the draft genome of strain P25^T^ revealed the presence of one copy each of *linA*, *linH*, *linK*, *linL*, *linM, linN*, and *linX*, and the IS FINDER database (13) (http://www-is.biotoul.fr) predicted the occurrence of IS*6* (*n* = 21), IS*1380* (*n* = 4), IS*3* (*n* = 1), and IS*256* (*n* = 1) as the major transposon families. However, *linB*, which encodes haloalkane dehalogenase, was absent, indicating that this strain has yet to acquire *linB* through horizontal gene transfer.

In comparison with the whole-genome sequence of *S. japonicum* UT26 (12), P25^T^ showed the presence of phenol- and toluene-degrading gene clusters, whereas homogentisate-, chlorophenol-, and anthranilate-degrading pathways were clearly absent in *S. quisquiliarum* P25^T^. Reciprocal smallest distance (RSD) analysis (e value, 10^–15^; distance, 0.125) revealed that *S. quisquiliarum* P25^T^ and *S. japonicum* UT26 share 1,650 orthologous genes. Data and information assimilation from the complete genome of this species and a comparative analysis with other sphingomonad genomes (3) are under way to expand our understanding of HCH degradation, especially the rapid evolution and acquisition of *lin* genes in sphingomonads under HCH selection pressure.

### Nucleotide sequence accession number.

The draft genome sequence of *Sphingobium quisquiliarum* P25^T^ has been deposited in GenBank under the accession number ATHO00000000. The version described in this paper is the first version.
